# Absorption of Drugs through the Skin

**Published:** 1908-03-28

**Authors:** 


### Medicine.

### ABSORPTION OF DRUGS THROUGH THE SKIN.

It has always been a very much discussed point as to how much therapeutic effect can be attained by the administration of drugs to patients through the skin. The teaching of physiologists was, for a long time, that little or nothing could be absorbed into the system through the unbroken skin, or, at any rate, through a normal skin, unless so much rubbing had been employed that the surface layers were to all intents and purposes denuded.

Clinical teaching, on the other hand, has always allowed that a considerable degree of drug-absorption can occur through unbroken epidermis: a very good example of the practical application of this belief is seen in the mercurial treatment of syphilis either by inunction or by fumigation. There is much clinical evidence to show that other oily substances can be absorbed in a similar way. The great improvement that can so often be noted in marasmic children when their bodies are anointed with cod-liver oil daily can scarcely be entirely due to diminution in the heat lost from the surface of their oily bodies. Heat loss can be inhibited in other ways—by clothing and by fires, and so forth —yet the rate of improvement brought about thus is nothing like so great as is that which results from gently rubbing oil into the skin.

### Absorption of Carbolic Acid.

That it does not take any particular friction of the skin surface to cause substances upon its outside to pass through into the vessels beneath is well shown by an unusual case reported from the Coroner's Court in January last. Mr. C. Luxmoore Drew held an inquest at Chelsea concerning the death of a man aged fifty-six, a butler at a house in Chelsea Embankment, death having occurred under strange circumstances. The report was that the man was found sitting in a bath partly filled with water into which carbolic acid had been poured. On a chair near at hand there was a bottle containing about half a pint of carbolic acid. The sufferer was not dead when he was found in the bath, but he was *in extremis*, and although artificial respiration was tried for some while, he died in about half an hour.

A post-mortem examination was made, and the viscera were reported to be perfectly sound. The doctor stated that, in his opinion, death was due to syncope from carbolic-acid poisoning. As there was no sign of any of the acid having been swallowed, the conclusion come to was that a lethal quantity had been absorbed through the skin. It appeared that the man was in the habit of taking these carbolic-acid baths, imagining that he had a skin disease.

This is the case in brief, as reported in the papers. It may be urged that there is no proof that there had been actual absorption of carbolic acid, but that fatal syncope had occurred when the patient happened to be in a carbolic-acid bath. Against this supposition there was the evidence that the man had no visceral disease, and there appeared to be no reason, except the carbolic acid, why he should have died. If not actual proof, yet the case affords presumptive evidence in favour of there having been carbolic-acid absorption through the skin.

### Salicylate Absorption.

The following is a more conclusive case in proof of the readiness with which at least some drugs may be absorbed into the system through the skin. The patient was a very intelligent man, who had the misfortune to suffer much from chronic rheumatism. Various remedies for the latter were tried, and amongst them salicylate of soda. This would relieve the joint pains, but never failed to make the man almost stone deaf. If ten grains of the salicylate were taken upon a single day, the deafness came on with certainty, and it lasted for nearly a fortnight sometimes, even when no further dose of salicylate was taken. This was such a nuisance to him that he was obliged to forego the use of the drug, and he put up with the joint pains rather than take the remedy with its untoward consequence.

One day, being particularly troubled with pains in his knee joint, he decided that, though he would take nothing in the form of salicylate by the mouth, he would apply a salicylate remedy to the joint locally; and he used an ordinary small quantity of betul oil, or oil of sweet birch, which contains salts of methyl-salicylic acid. The effect upon his hearing was identical with what would have been the result had salicylate been taken by the mouth. Within a few hours of the application to the knee the hearing began to go; next day the patient was stone-deaf, and remained so for nearly a fortnight.

It was clear, therefore, that quantities of salicylate had been absorbed through the skin round the knee, out of the betul oil.

### Double Conduction in Gland Tubules.

It is needless to multiply examples of such trans-epidermal drug absorption, but it is interesting to inquire into the process by which the absorption takes place.

Some work done upon surgical cases by, we believe, Mr. Bond of Leicester, throws a great deal of light upon the matter. This surgeon took the trouble, whenever he had to operate upon anything connected with a tubular structure in the body, to place some readily identifiable substance, such as indigo or carmine, at the wrong end of the duct or tube just before the operation, and then noted whether any of it had travelled up the tube the wrong way. For example, if an operation were to be done upon the cæcum he would place the carmine or other substance in the rectum; if a breast were to be amputated, he would apply some of the colouring matter to the nipple; and so on. He was thus able to show that in every case the colouring matter ascended the tube in which the main normal movement of the contents was downwards. The colouring matter ascended the colon from the rectum; ascended the ureters and reached the kidneys from the bladder; ascended the mammary ducts and entered into the substance of the gland, and even reached the lymphatics in the axilla; and so on. In other words, it was demonstrated in surgical cases in man that the ducts of glands, and tubes like the bowel, had both an out-going and an in-passing current, and that substances were constantly passing along them either way.

The significance of this in connection with ascending nephritis or surgical kidney might well be dilated upon; but at the present moment it is our purpose to discuss the bearing of the facts upon absorption through the skin. If there is both an ascending and a descending flow in big glandular ducts, it is reasonable to suppose that the same holds good for the ducts of little glands. The skin is full of these; there are both the sweat glands and the sebaceous glands; and even if only quite a small quantity of an application ascended into each duct, the total amount that would thereby reach a comparatively close contact with the blood-vessels of the corium might be considerable. It is doubtless in this way that drug absorption through the skin takes place.

